# Simulated datasets for population dynamics of sickle cell anaemia

**DOI:** 10.1016/j.dib.2017.12.006

**Published:** 2017-12-13

**Authors:** S.O. Edeki, O.O. Akanbi

**Affiliations:** a Department of Mathematics, Covenant University, Ota, Nigeria; b Department of Mathematics & Statistics, Federal Polytechnic Ilaro, Ilaro, Nigeria

**Keywords:** Sickle cell anaemia, Population dynamics, Data simulation

## Abstract

The datasets contained in this article are simulated data with respect to Sickle Cell Anaemia (SCA) in order to examine the mathematical inheritance formation of the SCA disease. The simulation is done using Monte Carlos Simulation (MCS) Technique to complement the Physical Simulation Smith's Statistical (PSSS) package used as random number generator for birth simulation. One hundred and fifty-six (156) births for seven (7) generations were considered in the simulation alongside non-gestating reproductive females with fertile male adults while immigration and emigration are not permitted. These datasets can effectively serve as benchmarks for both health, and marital counselling institutions.

**Specifications Table**

**Table:** 

Subject area	*Biomathematics*
More specific subject area	*Genetics, Sickle Cells.*
Type of data	*Table, Excel file.*
How data was acquired	*Data simulation via beads of two colours.*
Data format	*Analysed, CSV comma delimited.*
Experimental factors	Investigation of the genetics of sickle cell trait via mathematical simulation.
Experimental features	*Non-gestating reproductive female with fertile male adults.*
Data source location	Research Laboratory, Nigeria.
Data accessibility	*Within this article.*

**Value of the data**

• The dataset provided in this article reflects the usefulness of the concept of Monte Carlo technique in determining the population of sickle cell anaemia at any point in time.
• The dataset encourages the importance of genotype screening before marriage.
• The finiteness nature of the dataset can be used for estimating the sickle cell anaemia population statistic: mean frequencies based on the mutation rate.

## 1 Data

• The datasets used in this work are Sickle Cell Anaemia simulated data described in detail in [1]. This include the information contained in the Supplementary file. For related work on SCA, the following are referred [2], [3], [4], [5], [6], [7], [8].
• In addition, Table 1 shows the frequency of the genotype (***AA, AS, SS***), Table 2 contains the genotype cumulative probability and Tag-numbers, while Table 3 shows the birth results from different mating.

**Table 1:** Genotype frequency.

**Genotype**	**Frequency**
AA	69%
AS	28%
SS	3%

**Note:** During the physical simulation the birth of different genotypic group varied considerably with the distribution below.

**Table 2:** Genotype cumulative probability & Tag-numbers.

**Genotype**	**Probability**	**Cumulative probability**	**Tag–Numbers**
**AA**	0.69	0.69	0–68
**AS**	0.28	0.97	69–96
**SS**	0.03	1.00	97 -

**Table 3:** Birth results from different mating.

**Genotype**	**No of Birth**						
	1st gen./trial	2nd gen./trial	3rd gen./trial	4th gen./trial	5th gen./trial	6th gen./trial	7th gen./trial
AA	107	98	106	97	110	114	107
AS	47	55	47	55	41	39	43
SS	2	3	3	4	5	3	6

*Note:* gen./trial denotes generation per trial.
• Every concerned person is entitled to two copies of the gene which decides whether that person has Sickle Cell Anaemia or not. If both copies are “normal alleles” then only normal haemoglobin is produced that implies “**AA**”. If one of the two alleles is defective then that person has a mixture of normal and Sickle haemoglobin resulting to a condition known as Sickle Cell trait “**AS**” (Carrier). On the other hand, if both alleles are defective, then that person has Sickle Cell Anaemia referred to as “**SS**”.

## 2 Experimental design, materials and methods

Simulation has been recorded to have made life more physical. Based on a simulated annealing procedure and experimental observations. Mathematical models of heredity are to a greater extent based on one-locus, two allele genes population, where little or no attempt is made to consider the dynamics of the population by Monte Carlo simulation technique.

### 2.1 Methodology and data analysis

The method used in the data analysis of the different genotypic groups viz: **AA, AS, SS** is MCS whose detailed steps and procedures are contained in [1].
