# Supplementary material for: Simulated datasets for population dynamics of sickle cell anaemia
Source: Data Brief. 2017 Dec 13;16:876–9. doi: 10.1016/j.dib.2017.12.006 (PMC5847619; doi:10.1016/j.dib.2017.12.006)
Supplement: Supplementary file 2 — Supplementary material [file mmc2.pdf]

Supplementary I: Genotype Simulated Data

Couple's Number	Random Numbers							Genotype						
	1st $^{Gen}/_{trial}$	2nd $^{Gen}/_{trial}$	3rd $^{Gen}/_{trial}$	4th $^{Gen}/_{trial}$	5th $^{Gen}/_{trial}$	6th $^{Gen}/_{trial}$	7th $^{Gen}/_{trial}$	1st $^{Gen}/_{trial}$	2nd $^{Gen}/_{trial}$	3rd $^{Gen}/_{trial}$	4th $^{Gen}/_{trial}$	5th $^{Gen}/_{trial}$	6th $^{Gen}/_{trial}$	7th $^{Gen}/_{trial}$
1.	59	42	4	97	70	42	3	AA	AA	AA	SS	AS	AA	AA
2.	80	66	98	82	88	63	82	AS	AA	SS	AS	AS	AA	AS
3.	2	98	3	74	36	56	29	AA	SS	AA	AS	AA	AA	AA
4.	7	43	66	12	29	49	15	AA	AA	AA	AA	AA	AA	AA
5.	52	0	24	74	94	3	31	AA	AA	AA	AS	AS	AA	AA
6.	73	37	49	10	58	46	77	AS	AA	AA	AA	AA	AA	AS
7.	49	31	11	54	65	35	48	AA	AA	AA	AA	AA	AA	AA
8.	89	64	99	7	44	68	8	AS	AA	SS	AA	AA	AA	AA
9.	76	75	45	63	86	44	70	AS	AS	AA	AA	AS	AA	AS
10.	54	90	16	13	0	8	77	AA	AS	AA	AA	AA	AA	AS
11.	94	50	17	64	6	86	8	AS	AA	AA	AA	AA	AS	AA
12.	92	94	35	68	29	67	30	AS	AS	AA	AA	AA	AA	AA
13.	40	47	79	70	26	78	36	AA	AA	AS	AS	AA	AS	AA
14.	95	42	31	57	44	53	11	AS	AA	AA	AA	AA	AA	AA
15.	16	48	53	35	49	70	57	AA	AA	AA	AA	AA	AS	AA
16.	14	36	5	48	11	10	79	AA	AA	AA	AA	AA	AA	AS
17.	26	20	39	24	5	8	15	AA	AA	AA	AA	AA	AA	AA
18.	19	80	71	40	80	7	89	AA	AS	AS	AA	AS	AA	AS
19.	54	0	34	32	30	88	19	AA	AA	AA	AA	AA	AS	AA
20.	28	76	71	1	43	9	56	AA	AS	AS	AA	AA	AA	AA
21.	66	10	86	25	90	33	27	AA	AA	AS	AA	AS	AA	AA
22.	73	77	69	65	21	77	13	AS	AS	AS	AA	AA	AS	AA
23.	67	87	88	29	87	20	69	AA	AS	AS	AA	AS	AA	AS

24.	8	66	39	49	26	9	88	AA	AA	AA	AA	AA	AA	AS
25.	16	44	54	82	29	35	70	AA	AA	AA	AS	AA	AA	AS
26.	82	35	70	6	63	2	77	AS	AA	AS	AA	AA	AA	AS
27.	59	82	30	64	33	67	93	AA	AS	AA	AA	AA	AA	AS
28.	34	70	5	45	76	23	67	AA	AS	AA	AA	AS	AA	AA
29.	38	82	44	38	10	55	21	AA	AS	AA	AA	AA	AA	AA
30.	61	17	34	43	13	16	8	AA	AA	AA	AA	AA	AA	AA
31.	93	93	31	99	76	23	29	AS	AS	AA	SS	AS	AA	AA
32.	0	23	39	47	47	61	71	AA	AA	AA	AA	AA	AA	AS
33.	61	90	98	31	37	82	12	AA	AS	SS	AA	AA	AS	AA
34.	3	27	32	23	11	23	97	AA	AA	AA	AA	AA	AA	AS
35.	62	92	75	30	85	52	85	AA	AS	AS	AA	AS	AA	AS
36.	35	40	9	23	56	81	19	AA	AA	AA	AA	AA	AS	AA
37.	17	9	8	62	32	56	69	AA	AA	AA	AA	AA	AA	AS
38.	46	6	59	25	18	90	4	AA	AA	AA	AA	AA	AS	AA
39.	25	8	95	20	31	6	12	AA	AA	AS	AA	AA	AA	AA
40.	56	36	70	76	54	0	40	AA	AA	AS	AS	AA	AA	AA
41.	38	80	91	73	44	48	46	AA	AS	AS	AS	AA	AA	AA
42.	14	95	9	28	83	0	43	AA	AS	AA	AA	AS	AA	AA
43.	88	98	29	49	76	75	10	AS	SS	AA	AA	AS	AS	AA
44.	62	82	87	44	78	59	53	AA	AS	AS	AA	AS	AA	AA
45.	65	18	56	57	4	64	48	AA	AA	AA	AA	AA	AA	AA
46.	77	63	67	69	49	44	23	AS	AA	AA	AS	AA	AA	AA
47.	88	81	91	37	61	35	56	AS	AS	AS	AA	AA	AA	AA
48.	38	21	1	91	4	69	91	AA	AA	AA	AS	AA	AS	AS
49.	67	60	70	13	43	9	86	AA	AA	AS	AA	AA	AA	AS
50.	24	45	73	89	80	51	76	AA	AA	AS	AS	AS	AA	AS

51.	33	60	40	0	37	71	23	AA	AA	AA	AA	AA	AS	AA
52.	0	11	64	68	70	50	93	AA	AA	AA	AA	AS	AA	AS
53.	88	7	7	46	99	18	97	AS	AA	AA	AA	SS	AA	SS
54.	78	64	35	34	5	71	86	AS	AA	AA	AA	AA	AS	AS
55.	94	68	47	60	93	98	19	AS	AA	AA	AA	AS	SS	AA
56.	68	57	42	77	1	53	24	AA	AA	AA	AS	AA	AA	AA
57.	39	87	38	75	28	82	31	AA	AS	AA	AS	AA	AS	AA
58.	29	72	68	91	85	26	44	AA	AS	AA	AS	AS	AA	AA
59.	39	5	59	52	51	4	34	AA	AA	AA	AA	AA	AA	AA
60.	49	2	27	86	6	12	78	AA	AA	AA	AS	AA	AA	AS
61.	66	63	25	52	90	72	65	AA	AA	AA	AA	AS	AS	AA
62.	63	16	59	82	93	46	72	AA	AA	AA	AS	AS	AA	AS
63.	94	31	22	97	78	47	75	AS	AA	AA	SS	AS	AA	AS
64.	11	79	19	89	26	88	20	AA	AS	AA	AS	AA	AS	AA
65.	5	47	63	11	28	85	65	AA	AA	AA	AA	AA	AS	AA
66.	46	71	31	28	27	59	10	AA	AS	AA	AA	AA	AA	AA
67.	81	25	20	79	93	50	66	AS	AA	AA	AS	AS	AA	AA
68.	18	10	9	32	46	13	66	AA	AA	AA	AA	AA	AA	AA
69.	5	78	67	94	9	54	74	AA	AS	AA	AS	AA	AA	AS
70.	5	3	8	69	12	39	97	AA	AA	AA	AS	AA	AA	SS
71.	27	9	71	91	76	39	39	AA	AA	AS	AS	AS	AA	AA
72.	94	75	41	96	80	37	86	AS	AS	AA	AS	AS	AA	AS
73.	42	54	69	14	64	42	27	AA	AA	AS	AA	AA	AA	AA
74.	58	37	74	72	85	77	15	AA	AA	AS	AS	AS	AS	AA
75.	87	18	21	26	49	46	84	AS	AA	AA	AA	AA	AA	AS
76.	28	57	16	1	82	71	90	AA	AA	AA	AA	AS	AS	AS
77.	47	86	5	98	55	48	54	AA	AS	AA	SS	AA	AA	AA

78.	81	38	25	41	86	18	24	AS	AA	AA	AA	AS	AA	AA
79.	89	11	84	96	65	95	63	AS	AA	AS	AS	AA	AS	AA
80.	37	13	42	6	29	81	23	AA	AA	AA	AA	AA	AS	AA
81.	20	51	82	90	94	77	46	AA	AA	AS	AS	AS	AS	AA
82.	46	70	41	85	30	71	74	AA	AS	AA	AS	AA	AS	AS
83.	14	69	61	96	86	61	48	AA	AS	AA	AS	AS	AA	AA
84.	70	90	69	80	99	52	65	AS	AS	AS	AS	SS	AA	AA
85.	85	78	46	96	5	69	97	AS	AS	AA	AS	AA	AS	SS
86.	93	15	7	70	11	40	61	AS	AA	AA	AS	AA	AA	AA
87.	2	57	38	73	33	31	47	AA	AA	AA	AS	AA	AA	AA
88.	86	65	23	69	76	66	27	AS	AA	AA	AS	AS	AA	AA
89.	36	95	85	58	91	94	6	AA	AS	AS	AA	AS	AS	AA
90.	74	54	33	64	54	11	15	AS	AA	AA	AA	AA	AA	AA
91.	64	39	37	84	36	69	64	AA	AA	AA	AS	AA	AS	AA
92.	2	89	12	3	57	17	83	AA	AS	AA	AA	AA	AA	AS
93.	86	36	53	88	1	28	43	AS	AA	AA	AS	AA	AA	AA
94.	47	84	35	57	0	20	62	AA	AS	AA	AA	AA	AA	AA
95.	14	14	71	65	3	15	15	AA	AA	AS	AA	AA	AA	AA
96.	28	58	42	2	69	67	33	AA	AA	AA	AA	AS	AA	AA
97.	99	93	31	75	21	58	63	SS	AS	AA	AS	AA	AA	AA
98.	88	52	22	43	88	11	48	AS	AA	AA	AA	AS	AA	AA
99.	30	86	71	38	10	26	35	AA	AS	AS	AA	AA	AA	AA
100.	55	11	77	11	36	47	32	AA	AA	AS	AA	AA	AA	AA
101.	68	30	71	63	20	84	76	AA	AA	AS	AA	AA	AS	AS
102.	8	71	52	92	91	8	8	AA	AS	AA	AS	AS	AA	AA
103.	89	82	40	86	29	74	69	AS	AS	AA	AS	AA	AS	AS
104.	16	88	75	11	98	83	51	AA	AS	AS	AA	SS	AS	AA

105.	90	76	78	70	45	68	38	AS	AS	AS	AS	AA	AS	AA
106.	29	83	83	57	80	65	78	AA	AS	AS	AA	AS	AA	AS
107.	44	56	91	86	21	75	38	AA	AA	AS	AS	AA	AS	AA
108.	86	76	10	28	71	82	29	AS	AS	AA	AA	AS	AS	AA
109.	60	98	90	0	10	22	12	AA	SS	AS	AA	AA	AA	AA
110.	21	87	71	32	39	16	86	AA	AS	AS	AA	AA	AA	AS
111.	37	39	92	42	12	55	11	AA	AA	AS	AA	AA	AA	AA
112.	80	0	41	9	90	20	13	AS	AA	AA	AA	AS	AA	AA
113.	22	90	42	24	38	52	53	AA	AS	AA	AA	AA	AA	AA
114.	96	46	63	22	98	26	65	AS	AA	AA	AA	SS	AA	AA
115.	69	36	19	10	92	83	28	AS	AA	AA	AA	AS	AS	AA
116.	35	15	39	13	74	23	63	AA	AA	AA	AA	AS	AA	AA
117.	5	66	43	47	40	98	33	AA	AA	AA	AA	AA	SS	AA
118.	11	47	93	52	56	55	27	AA	AA	AS	AA	AA	AA	AA
119.	56	80	72	86	54	15	58	AA	AS	AS	AS	AA	AA	AA
120.	74	19	12	57	17	19	67	AS	AA	AA	AA	AA	AA	AA
121.	64	66	39	95	63	26	42	AA	AA	AA	AS	AA	AA	AA
122.	93	93	92	8	33	68	20	AS	AS	AS	AA	AA	AA	AA
123.	46	22	36	81	59	35	59	AA	AA	AA	AS	AA	AA	AA
124.	78	56	17	31	35	92	53	AS	AA	AA	AA	AA	AS	AA
125.	60	78	6	96	61	44	77	AA	AS	AA	AS	AA	AA	AS
126.	31	9	60	14	84	70	34	AA	AA	AA	AA	AS	AS	AA
127.	84	37	79	68	64	67	62	AS	AA	AS	AA	AA	AA	AA
128.	76	49	35	11	43	21	31	AS	AA	AA	AA	AA	AA	AA
129.	29	81	82	39	75	30	42	AA	AS	AS	AA	AS	AA	AA
130.	37	26	21	89	35	83	86	AA	AA	AA	AS	AA	AS	AS
131.	10	79	13	75	48	20	12	AA	AS	AA	AS	AA	AA	AA

132.	20	4	26	75	50	9	15	AA	AA	AA	AS	AA	AA	AA
133.	89	50	71	75	4	25	97	AS	AA	AS	AS	AA	AA	SS
134.	69	29	20	47	46	46	19	AS	AA	AA	AA	AA	AA	AA
135.	63	83	76	94	39	51	51	AA	AS	AS	AS	AA	AA	AA
136.	96	67	62	34	9	43	27	AS	AA	AA	AA	AA	AA	AA
137.	44	66	94	47	18	20	64	AA	AA	AS	AA	AA	AA	AA
138.	86	11	59	21	2	43	33	AS	AA	AA	AA	AA	AA	AA
139.	55	87	69	97	48	48	82	AA	AS	AS	AS	AA	AA	AS
140.	17	88	41	92	1	37	45	AA	AS	AA	AS	AA	AA	AA
141.	4	73	62	68	45	72	82	AA	AS	AA	AA	AA	AS	AS
142.	60	71	84	19	52	98	70	AA	AS	AS	AA	AA	SS	AS
143.	9	53	80	87	43	45	94	AA	AA	AS	AS	AA	AA	AS
144.	8	83	56	82	42	39	45	AA	AS	AA	AS	AA	AA	AA
145.	74	62	68	52	14	76	87	AS	AA	AA	AA	AA	AS	AS
146.	99	3	72	42	40	60	40	SS	AA	AA	AA	AA	AA	AA
147.	21	53	48	29	38	50	51	AA	AA	AA	AA	AA	AA	AA
148.	54	2	10	4	27	35	14	AA	AA	AA	AA	AA	AA	AA
149.	12	59	3	72	3	54	39	AA	AA	AA	AS	AA	AA	AA
150.	66	60	86	86	25	5	85	AA	AA	AS	AS	AA	AA	AS
151.	88	68	25	27	22	39	41	AS	AA	AA	AA	AA	AA	AA
152.	66	17	16	63	49	57	56	AA	AA	AA	AA	AA	AA	AA
153.	29	75	25	96	98	85	55	AA	AS	AA	AS	SS	AS	AS
154.	82	77	87	83	4	15	99	AS	AS	AS	AS	AA	AA	SS
155.	7	45	24	1	16	89	96	AA	AA	AA	AA	AA	AS	AS
156.	38	73	24	4	71	6	85	AA	AS	AA	AA	AS	AA	AS
